# Genomic and Functional Characterization of Three Probiotic Strains with Features Relevant to Cholesterol and Choline-TMAO Metabolism: Rationale for a Cardiometabolic Formulation

**DOI:** 10.3390/genes17101214

**Published:** 2026-09-30

**Authors:** Annalisa Visciglia, Giovanni Deusebio, Angela Amoruso, Marco Pane

**Affiliations:** Probiotical Research Srl, 28100 Novara, Italy; a.visciglia@probiotical.com (A.V.); g.deusebio@probiotical.com (G.D.); m.pane@probiotical.com (M.P.)

**Keywords:** cardiovascular disease, cholesterol, probiotics, TMAO, gut microbiota, genomics, bile salt hydrolase

## Abstract

**Background/Objectives:** The gut microbiota acts on two cardiovascular risk factors that are open to intervention: circulating cholesterol and TMAO, which derives from bacterial conversion of dietary choline to trimethylamine. We characterised *Bifidobacterium breve* BR03 (DSM 16604), *Lactiplantibacillus plantarum* LP14 (DSM 33401) and *Limosilactobacillus reuteri* LRE11 (DSM 33827), candidate components of a cardiometabolic formulation. Genomic profiling across seven functional categories was combined with an *in vitro* assessment of cholesterol removal. **Methods:** Whole-genome screening was used to identify genes related to oxidative stress resistance, adhesion, cholesterol-related functions (including *bsh*), folate biosynthesis, immune-interface functions and degradation of anti-nutritional factors. In addition, an in vitro assay assessed cholesterol removal from the medium. **Results:** All three strains carry *bsh* at 90.7–100% identity, along with core adhesion determinants and antioxidant systems, and all three are genetically incapable of generating trimethylamine: neither *cutC* nor *cntA* is present in assemblies that are essentially complete (100% for LP14, 99.5% for LRE11, closed and optically validated for BR03). The three repertoires then diverge. LP14 provides near-complete folate biosynthesis, the broadest set of surface and immune-interface genes, a choline kinase homologue at 99.6% and putative matches to *choXWV*. BR03 contributes the widest range of enzymes degrading anti-nutritional factors. BR03 and LRE11 remove the most cholesterol in vitro. **Conclusions:** The three strains cover complementary metabolic and surface functions while sharing a safety-relevant feature: none can contribute to TMAO formation. This profile makes them suitable candidates for a combined cardiometabolic formulation, whose clinical application will be tested in a planned clinical study.

## 1. Introduction

Scientific research has progressively highlighted the critical role of the gut microbiome in modulating diverse physiological and pathological processes [1,2,3,4]. Among these, cardiovascular diseases (CVD) have emerged as a major area of investigation, with mounting evidence supporting a substantial contribution of gut microbial metabolites to CVD onset and progression [5,6]. A landmark discovery linked dietary phosphatidylcholine, a nutrient abundant in Western diets, with gut microbiota-dependent formation of trimethylamine N-oxide (TMAO), a metabolite now recognized as an independent predictor of adverse cardiovascular events [7,8]. TMAO is generated via a meta-organismal pathway that begins with microbial metabolism of dietary choline, phosphatidylcholine, and carnitine into trimethylamine (TMA), followed by hepatic oxidation of TMA to TMAO via flavin monooxygenases (FMOs) [9,10,11]. Given the ubiquity of choline in common dietary staples (e.g., eggs, red meat, fish, and some vegetables), as well as its endogenous presence in bile, microbial conversion of choline into TMA likely represents a major contributor to circulating TMAO levels across dietary patterns [12,13]. Mechanistically, TMAO promotes cardiovascular risk through several interconnected pathways, including disruption of cholesterol and sterol metabolism, activation of endothelial cells, promotion of vascular inflammation, stimulation of pro-fibrotic signaling cascades, and enhancement of vascular calcification via NLRP3 inflammasome and NF-κB activation [14,15,16,17]. TMAO also enhances platelet hyperreactivity by modulating calcium signaling, thereby increasing thrombosis propensity in animal models [18]. Clinical studies have confirmed that elevated TMAO levels correlate with increased platelet responsiveness and higher risk of major adverse cardiovascular events, such as myocardial infarction and stroke [19,20]. Interventional studies have shown that choline supplementation leads to increased TMAO production and platelet activation even in healthy individuals [21]. Beyond TMAO, the gut microbiota is known to influence host hemostasis and platelet biology by regulating factors such as serotonin, vitamin K, and von Willebrand factor [22,23,24]. These findings underscore the intricate link between microbial metabolism and cardiovascular pathophysiology. At the genetic level, microbial TMA production is catalyzed by the choline TMA lyase enzyme system, composed of the catalytic CutC protein and its activating partner CutD, encoded within the choline utilization (cut) gene cluster [25,26,27]. This pathway has emerged as a promising therapeutic target. For instance, the naturally occurring small molecule 3,3-dimethyl-1-butanol (DMB) has been shown to inhibit choline TMA lyase activity both in vitro and in vivo, reducing circulating TMAO levels and attenuating CVD phenotypes in animal models [28]. These insights have fueled growing interest in developing strategies to modulate gut microbiota-dependent TMAO production as a novel means to mitigate cardiometabolic risk [29,30]. In this context, probiotics have garnered attention for their potential to alter gut microbial community structure and function in favor of cardiovascular health [31,32]. Probiotics, defined as live microorganisms that, per the ISAPP consensus definition, confer a health benefit on the host when administered in adequate amounts, have been widely studied for their anti-inflammatory, lipid-lowering, and antioxidant properties [33,34,35,36]. In the setting of CVD, probiotics may attenuate TMAO-associated risk through multiple mechanisms: outcompeting TMA-producing taxa, enhancing gut barrier integrity, modulating host immunity and inflammation, and promoting beneficial metabolite production [37,38]. Certain strains may also interfere directly with microbial choline metabolism or secrete inhibitors of TMA lyase activity [39,40]. To identify effective probiotic candidates, functional genomics and whole-genome sequencing (WGS) are increasingly employed [41]. These approaches enable comprehensive strain characterization, allowing identification of genes involved in adhesion, bile salt hydrolase (*bsh*) activity (key in cholesterol metabolism), folate biosynthesis, antioxidant pathways, and immunomodulation [42,43,44,45,46]. This also facilitates safety assessment by verifying the absence of virulence factors and transferable antibiotic resistance genes [47,48]. Altogether, genomic insights provide a robust framework to rationally characterize candidate probiotic strains across multiple cardiometabolic-relevant axes, including lipid and bile-acid metabolism, folate provisioning, oxidative stress defenses, and choline/TMAO handling, and to evaluate the extent to which such genomic potential translates into measurable functional activity.

The functional categories examined here were selected to cover two complementary dimensions. Two are directly related to the cardiometabolic target: bile salt hydrolase activity, which affects cholesterol homeostasis, and the choline/TMA pathway, which determines whether a strain contributes to, or is neutral towards, TMAO formation. The remaining five (adhesion, degradation of anti-nutritional factors, folate biosynthesis, immunomodulation and oxidative stress response) address properties that determine whether a strain can persist and function in the intestinal environment and are therefore prerequisites for any cardiometabolic effect rather than incidental observations. The three strains examined here were pre-selected as the candidate components of a cardiometabolic formulation on the basis of species-level evidence and existing safety documentation. They represent three distinct genera commonly used in probiotic products, and the aim of this work was to establish whether, and to what extent, their genomic content supports the intended cardiometabolic rationale before proceeding to clinical evaluation.

## 2. Materials and Methods

### 2.1. Bioinformatic Investigation: Functional Analysis Through the Alignment of Predicted Genes Against a Protein Reference Database

An important computational step is to determine which genes are present, usually by aligning the translated DNA sequences against a reference database of protein sequences. We predicted genes from genomes of *Bifidobacterium breve* BR03 (DSM 16604), whose complete genome sequence has been previously described [49], *Lactiplantibacillus plantarum* LP14 (DSM 33401), and *Limosilactobacillus reuteri* LRE11 (DSM 33827) available in FASTA format, using PROKKA v1.14.6, a widely recognized tool for the annotation of prokaryotic genomes [50]. For each functional category, reference proteins were selected manually from the scientific literature on the basis of two criteria: the enzyme had to have a documented role in the pathway of interest, and a corresponding protein sequence had to be available in the NCBI database. Priority was given to sequences characterized in lactobacilli and bifidobacteria, which account for 219 of the 246 reference proteins used (89%); sequences from other genera were included only for enzymes with no characterized homologue in these taxa. The *choXWV* system was selected for analysis on the basis of its characterization in *Brucella* spp. [51]; the reference sequences used here derive from *Ligilactobacillus animalis* for *choX* and *choW*, and from *Brucella abortus* and *Pseudomonas* sp. for the ATP-binding component. The same reference database was used to query all three genomes, so that identity values are directly comparable across strains. The complete list of reference proteins, with accession numbers, protein descriptions and source organisms, is provided in Supplementary Table S1. We used DIAMOND v2.1.8 in blastx mode with the *--very-sensitive* setting to align predicted gene sequences against the reference protein database [52], applying an e-value threshold of 0.00001. Alignments were ranked by bit score and the best hit was retained for each gene. Reciprocal best-hit analysis, protein domain verification and genomic context inspection were not performed, and identity values are therefore reported as graded indicators rather than as confirmed orthology. Hits were retained when the alignment covered at least 60% of the reference sequence; no minimum identity threshold was applied at the filtering stage, and percent identity is reported for every retained hit in the corresponding tables. Because reference proteins were retrieved from heterogeneous bacterial species, identity values are interpreted as a graded indicator of sequence similarity rather than as a binary presence call: hits above 70% identity are described as confidently assigned, values between 30 and 70% as putative, and values below 30% as inconclusive and reported for completeness only. Values reported in the tables and heatmaps of Section 3.1 are therefore percent amino acid identity between the predicted protein of each strain and the corresponding reference protein, as returned by DIAMOND; they do not represent similarity between strains, and values in the same row are independent comparisons against a common reference rather than pairwise comparisons. Assembly and annotation statistics for the three genomes are provided in Supplementary Table S2. Genome completeness was assessed with BUSCO v6.1.0 using the lactobacillales_odb10 lineage dataset [53].

#### 2.1.1. Anti-Nutritional Factors (ANFs) Degradation-Related Genes

ANFs are compounds that interfere with the absorption or reduce the bioavailability of nutrients, and they are primarily found in plant-based foods. ANFs include indigestible non-starch polysaccharides, phytates (phytic acid), tannins, alkaloids, protease inhibitors (such as lectins and trypsin inhibitors), saponins, and others. These compounds can negatively impact nutrient uptake in the host organism. Certain bacterial enzymes, which are listed in Section 3.1, have been reported to be involved in the degradation of ANFs. Therefore, these enzymes were specifically searched for in the genomes of the bacterial strains analyzed in this study to assess their potential in breaking down ANFs and enhancing nutrient absorption.

#### 2.1.2. Adhesion-Related Genes

A selection of adhesion-related genes across three probiotic strains were investigated. These genes, listed in Section 3.1, are key for bacterial adhesion to host tissues, which is critical for the colonization and persistence of probiotics in the gastrointestinal tract. The genes analyzed include those encoding sortase enzymes, glycosyl transferases, and mucus-binding proteins, which facilitate interaction with the host mucosal surface. We classified adhesion-related genes into canonical surface-anchored markers (e.g., sortase/LPXTG, mucus-binding proteins) and so-called “moonlighting” enzymes (e.g., enolase, triosephosphate isomerase) that, despite primary cytosolic roles, can be displayed on the bacterial surface and mediate host-component binding.

#### 2.1.3. Cholesterol Reduction Gene

Probiotic strains may contribute to cholesterol reduction through several mechanisms, including bile salt deconjugation mediated by bile salt hydrolase (*bsh*), cholesterol binding to the cell surface, and co-precipitation with deconjugated bile acids. Various annotated versions of the *bsh* gene from different species were retrieved from NCBI and are listed in Section 3.1. These gene sequences were then searched within the genomes of the examined strains.

#### 2.1.4. Production of Folate-Related Genes

Probiotic strains are known for their ability to produce folates, essential B vitamins involved in key cellular functions. This capability is linked to the presence of genes encoding enzymes in the folate biosynthesis pathway, such as *folP*, *folC*, and *folE.* To investigate this potential, annotated sequences of these genes from different Lactobacillus species were retrieved from NCBI (Section 3.1) and subsequently screened within the genomes of the analyzed strains.

#### 2.1.5. Immunomodulation-Related Genes

Probiotic strains can influence the host immune response through the expression of immunomodulatory genes, which may play a role in cardiovascular health. Annotated sequences of these genes from various bacterial species were retrieved from NCBI (Section 3.1) and analyzed to determine their presence within the genomes of the selected strains [42].

#### 2.1.6. Oxidative Stress-Related Genes

Oxidative stress plays a crucial role in cardiovascular diseases, and certain probiotic strains possess mechanisms to counteract it. This protective effect is associated with genes encoding antioxidant enzymes, such as catalase and thioredoxin reductase, which help neutralize reactive oxygen species (ROS) and reduce cellular damage. Annotated sequences of these genes from various bacterial species were retrieved from NCBI (Section 3.1) and screened within the genomes of the analyzed strains to assess their potential antioxidant capacity.

#### 2.1.7. Choline Uptake and Conversion Genes

Genes involved in the microbial transformation of choline into phosphatidylcholine, as well as those responsible for its uptake and undesired conversion into trimethylamine (TMA), were investigated. Choline can be metabolized via the CDP-choline pathway through a series of enzymatic steps involving choline kinase (CK), phosphocholine cytidylyltransferase (PCYT), and cholinephosphotransferase (CPT), ultimately leading to the formation of phosphatidylcholine, a membrane phospholipid with anti-inflammatory properties. In contrast, certain bacterial genes, namely *cutC* (choline TMA-lyase), *cutD* (its activating enzyme), and *cntA* (carnitine monooxygenase subunit), are responsible for the conversion of choline or carnitine into TMA, a precursor of proatherogenic trimethylamine-N-oxide (TMAO). The presence of these genes was screened to ensure the safety and metabolic neutrality of the selected strains. In addition, genes related to the active transport of choline, including the high-affinity transporter complex *choXWV*, were also searched [52]. All genes mentioned above are listed in Section 3.1 and were specifically sought in the bacterial genomes analyzed in this study to assess their potential role in choline utilization and to exclude strains harboring proatherogenic traits. Reference sequences for *choX* and *choW* derive from *L. animalis*, while the ATP-binding component references derive from *B. abortus* and *Pseudomonas* sp.; identity values should be interpreted accordingly.

### 2.2. Cholesterol Reduction Investigation

Probiotic strains BR03, LRE11 and LP14, previously screened by bioinformatic analysis, were examined to investigate their ability to reduce cholesterol levels. Strains were inoculated (1% *v*/*v*) in MRS supplemented with cholesterol-PEG 600 (0.1 g/L) and incubated at 37 °C for 16 ± 2 h. After incubation, all cultures were counted through flow cytometry and centrifuged (4000 rpm–10 min). Supernatants were collected and tested for the presence of Cholesterol-by-Cholesterol Quantification Assay Kit (cod. CS0005; Sigma-Aldrich, St. Louis, MO, USA). Cholesterol concentration in each supernatant was determined from a standard curve prepared in MRS broth (Difco, BD Biosciences, Franklin Lakes, NJ, USA), to account for matrix effects of the growth medium, and expressed per mL of culture. The calibration curve covered the range 0–5 µg cholesterol per well (0–100 µg/mL), with the regression equation A = 0.0318·C + 0.3476 and R^2^ = 0.9925. Viable cell counts were determined by flow cytometry on a NovoCyte (ACEA Biosciences, San Diego, CA, USA) instrument using the BD Cell Viability Kit (BD Biosciences, Franklin Lakes, NJ, USA), with thiazole orange (TO) and propidium iodide (PI) as the viability dyes; events positive for TO and negative for PI were counted as viable (alive fluorescent units, AFU). Cholesterol reduction was calculated as the difference between the uninoculated substrate reference (MRS supplemented with cholesterol-PEG 600 at 0.1 g/L C 5670; Sigma-Aldrich, St. Louis, MO, USA) and each strain supernatant, and normalized to the viable cell density, giving cholesterol removed per 10^9^ viable cells (µg per 10^9^ AFU). An uninoculated control incubated in parallel was also included. The assay quantifies cholesterol disappearance from the supernatant and does not distinguish metabolic transformation from adsorption to the bacterial surface or removal with the cell pellet during centrifugation. Viable counts were determined at the end of the incubation and used as the normalization factor. This denominator represents the biomass present at sampling rather than cumulative cell exposure over the growth period, and normalized values should therefore be read as removal per unit of final viable biomass. Flow-cytometric AFU and plate-count CFU are not equivalent measurements, since flow cytometry enumerates cells that are membrane-intact by TO/PI staining whereas CFU counts culturable cells; the two can differ appreciably, and the values reported here refer to AFU only.

## 3. Results

### 3.1. Bioinformatic Analysis

#### 3.1.1. ANFs Degradation-Related Genes

The data showed moderate-to-high sequence identity between the probiotic genomes and selected ANF degradation-related reference genes, as illustrated in Figure 1 and Table 1. Notably, strains BR03 and LP14 exhibited a strong genetic correspondence, particularly for genes involved in the production of protease and alpha galactosidase. This high alignment suggests that strains may play a significant role in breaking down anti-nutritional factors as well as improving the digestion of complex carbohydrates through alpha-galactosidase activity.

#### 3.1.2. Adhesion-Related Genes

The analysis revealed that all three probiotic strains showed sequence matches to multiple adhesion-related genes, with LP14 displaying the broadest repertoire and the highest number of high-identity hits, as illustrated in Figure 2 and Table 2. Overall, core adhesion-associated genes (triosephosphate isomerase, enolase, glycosyl transferase, ATP synthase ε) were confidently assigned in all three strains, whereas the mucus-binding protein matched only at putative identity in LRE11 and LP14 and was not detected in BR03.

#### 3.1.3. Cholesterol Reduction-Related Genes

The analysis, as shown in Figure 3 and Table 3, revealed that all three probiotic strains possess the *bsh* gene, with sequence identity to the best-matching reference sequence in each case ranging from 90.7% (BR03) to 100% (LRE11) and 99.7% (LP14), supporting a role for these strains in cholesterol reduction. Because these reference sequences were derived from different species (see Section 2.1.3), and because the best hit corresponded to a different reference protein for each strain, percent identity is not directly comparable across strains and should not be read as a ranking of functional potency; the corresponding functional data are presented in Section 3.2 and interpreted alongside this caveat in the Discussion.

#### 3.1.4. Production of Folate-Related Genes

Among the analyzed strains, LP14 exhibited the highest potential for folate biosynthesis, as it contained nearly all the key genes involved in the folate production pathway. Specifically, genes such as *folC*, *folE*, *trpE*, *trpG* and *dfrA* were identified within its genome, suggesting a more complete genomic potential for folate biosynthesis compared with the other strains. In contrast, the other strains showed an incomplete gene set, potentially limiting their folate-producing capacity, as shown in Figure 4 and Table 4.

#### 3.1.5. Immunomodulation-Related Genes

Analyzing the presence of genes associated with immune-interface functions, the results revealed that strain LP14 exhibited the highest correspondence with the genes analyzed, with nearly all of them present within its genome (Figure 5 and Table 5). In most cases where LP14 lacked a specific gene, at least one of the other strains possessed it, although a few loci were matched in a single strain only and at low identity. Most of these genes encode surface-associated structures (exopolysaccharide and teichoic-acid machinery) and bacteriocin systems of the *pln* cluster rather than direct effectors of host immunity; their presence therefore indicates a capacity to engage innate immune interfaces across the formulation, rather than demonstrated immunomodulation.

#### 3.1.6. Oxidative Stress-Related Genes

The analysis of oxidative stress-related genes revealed that the Lactobacillus strains, particularly LP14, showed the highest presence of genes encoding antioxidant enzymes such as catalase and thioredoxin reductase, which are crucial for neutralizing reactive oxygen species (ROS) and reducing cellular damage, as shown in Figure 6 and Table 6. Confident assignments were largely restricted to LP14; in BR03 and LRE11 several of the transporters and reductases examined fell in the inconclusive range and are reported for completeness only. Given that oxidative stress is a well-established risk factor for cardiovascular diseases (CVD), this gene profile supports a potential antioxidant capacity, particularly for LP14, although functional redox assays are required to confirm activity.

#### 3.1.7. Choline Uptake and Conversion Genes

No homologues of *cutC* or *cntA* were detected in any of the three genomes under the search criteria applied (Figure 7, Table 7). Genome completeness was 100% for LP14 and 99.5% for LRE11 (BUSCO, lactobacillales_odb10; Supplementary Table S2), and the BR03 assembly is a closed genome validated by optical mapping, indicating that these absences are unlikely to reflect incomplete assemblies. Notably, LP14 carried *cutD*, the CutC-activating enzyme encoded within the same operon, at full sequence identity, indicating that the search was able to detect members of this locus; the absence of a *cutC* hit is therefore unlikely to reflect insufficient sensitivity of the alignment approach. This profile is consistent with a low intrinsic potential for TMA production. None of the strains possess the full pathway for converting choline into phosphatidylcholine; however, LP14 returned matches to the three components of the choline uptake system *choXWV*, at identities of 52.8%, 68.6% and 50.0% respectively. All three fall within the putative range defined in Section 2.1, so the presence of a functional choline-uptake system in this strain remains tentative. The functional implications of this profile are addressed in Section 4 (Discussion).

### 3.2. Cholesterol Reduction

All three probiotic strains reduced cholesterol from the medium (Table 8). Reductions differed by strain with BR03 largest and most reproducible, LP14 smallest, and LRE11 highly variable. Values are expressed as cholesterol removed per 10^9^ viable cells (µg per 10^9^ AFU), normalized to flow-cytometric viable counts to account for differences in growth between strains. Because the medium was not supplemented with conjugated bile salts, bile salt hydrolase cannot act under these conditions. The assay therefore measures net cholesterol removal by mechanisms that do not require *bsh* activity, such as binding to the cell surface and incorporation into the bacterial membrane, and it does not test the function of the *bsh* gene detected in the three genomes. The corresponding t-based 95% confidence intervals (*n* = 3), computed from the reported summary statistics, are BR03 [391, 462], LRE11 [−100, 761] and LP14 [8, 30] µg per 10^9^ AFU; the LRE11 interval is wide and spans zero, indicating substantial uncertainty around its point estimate, and a summary-statistics comparison distinguishes BR03 from LP14 (*p* ≈ 0.0001) but not LRE11 from LP14 (*p* ≈ 0.09).

## 4. Discussion

In this work, we combined whole-genome screening with an in vitro cholesterol-reduction assay to characterize three probiotic strains, *B. breve* BR03 (DSM 16604), *L. reuteri* LRE11 (DSM 33827), and *L. plantarum* LP14, for features relevant to cardiometabolic health: adhesion, bile salt hydrolase carriage, folate biosynthesis, oxidative stress defenses, degradation of anti-nutritional factors (ANFs), and choline utilization. These genomic data are hypothesis-generating rather than confirmatory: gene presence indicates metabolic capacity, not expression or activity in vivo, and the profiles below are interpreted on that basis. Read this way, the three strains show complementary rather than overlapping roles, with LP14 contributing breadth across metabolic, adhesive, and immune-interface functions and BR03 and LRE11 showing greater in vitro cholesterol removal, which is the rationale for combining them in a single formulation. All three strains carried the *bsh* gene and, in a separate assay, reduced cholesterol from a medium lacking conjugated bile salts. These are two independent observations: the genomic finding is relevant to bile-acid deconjugation in the intestinal environment, where conjugated substrates are available, whereas the in vitro reduction reflects *bsh*-independent mechanisms. The magnitude of reduction differed by strain: BR03 produced the most reproducible effect and the highest point estimate (426.8 ± 8.3 µg per 10^9^ AFU), LP14 the lowest (18.9 ± 2.55 µg per 10^9^ AFU), and LRE11 an intermediate but considerably more variable value (330.4 ± 100 µg per 10^9^ AFU), which we regard as active but in need of confirmation given the dispersion across the three replicates. We did not rank the strains by *bsh* sequence identity, as identity was scored against heterogeneous reference sequences and does not support activity comparisons between strains. The differences in reduction are more plausibly explained by strain physiology, including cell-surface composition, biomass yield and membrane lipid content, together with assay factors such as viable-cell density at sampling despite AFU normalization. Beyond lipid handling, the genomic profiles point to complementary mechanisms at the ecological and nutritional level. Genes linked to proteases and α-galactosidase, notably in BR03 and LP14, suggest a capacity to degrade ANFs and complex carbohydrates, with potential benefits for nutrient bioavailability and cross-feeding within the microbiota. Alongside these, canonical adhesion markers (e.g., sortase/LPXTG, mucus-binding proteins) and “moonlighting” adhesins (e.g., enolase, triosephosphate isomerase) are consistent with colonization competence, a prerequisite for sustained activity in vivo. LP14 carried the broadest accessory repertoire across adhesion, folate, and oxidative stress categories, indicating a strain with strong persistence potential and host-interactive metabolism. Durable mucosal association would be expected to increase local exposure of host tissues to strain-derived metabolites such as folate and to surface structures, while antioxidant pathways may limit the ROS amplification that contributes to vascular dysfunction. This provisioning potential is most evident in the folate pathway: LP14 encoded nearly the complete set of biosynthetic genes, including *folC*, *folE*, *trpE*, and *trpG*, indicating a higher intrinsic capacity for B-vitamin synthesis. Folate is relevant to endothelial function and one-carbon metabolism and, indirectly, to lipid and homocysteine homeostasis. Folate output was not quantified here, so the genomic completeness serves as a rationale for targeted metabolite assays rather than as evidence of production. A further axis relevant to cardiovascular risk concerns how the strains handle dietary choline. No *cutC* or *cntA* homologues were detected, indicating a low intrinsic potential for direct TMA generation. The presence of a choline kinase homologue in LP14, together with putative matches to the *choXWV* components, is compatible with, but does not demonstrate, a capacity to take up and consume choline. This is particularly so because the complete pathway for conversion of choline to phosphatidylcholine was not detected. Any competition with TMA-producing members of the microbiota therefore remains a hypothesis rather than a demonstrated effect: the absence of TMA-generating genes in these strains does not by itself prevent TMA production by other community members, and testing it will require co-culture or ex vivo assays with host TMAO measurements. The strains also carry genes with a potential defensive role against oxidative and inflammatory insults. LP14 showed an enriched antioxidant gene set, including catalase and thioredoxin machinery, which may buffer redox-sensitive inflammatory signaling implicated in vascular pathology. The genes we grouped under immune interaction are largely surface-associated (exopolysaccharide and teichoic-acid machinery) and bacteriocin systems of the pln cluster rather than direct effectors of host immunity; they are therefore better read as capacity to engage innate immune interfaces than as demonstrated immunomodulation. This capacity was distributed across the formulation rather than concentrated in one strain, consistent with a model in which the combination, rather than any single strain, would provide this capacity; this model has not been tested experimentally. Overall, the complementary contributions of the three strains, spanning lipid handling, colonization, folate provisioning, choline handling, and antioxidant and surface-immune functions, form the basis for evaluating them as a fixed combination rather than individually. No experiments were performed with the three strains in combination, and no data on compatibility, co-survival, antagonism or additive activity are presented here; these are the subject of the planned clinical evaluation. On this rationale, the three strains were selected for a prospective, single-centre, randomized, placebo-controlled, low-intervention clinical study in patients with coronary artery disease and concomitant peripheral arterial disease, assessing changes in vascular structure and function alongside predefined panels of endothelial, inflammatory, platelet, and microbiota-derived metabolite biomarkers. The composition, dose and duration of the intervention are specified in the study protocol and are outside the scope of the present characterization. The present manuscript provides the mechanistic rationale and the a priori selection criteria that informed that formulation, with the working hypothesis that the combination of bile salt hydrolase carriage, choline handling, folate provisioning, and antioxidant and surface-immune activity translates into measurable vascular and metabolic effects. Several limitations apply. Gene presence does not establish expression or activity in vivo, and functional confirmation through targeted metabolite, transcriptomic, or proteomic work is needed across all the axes discussed, not only cholesterol reduction. Our inference on the choline/TMAO axis is mechanistic and untested. The in vitro cholesterol assay was limited to a single condition and three replicates, with notable variability in the LRE11 estimate; *bsh* sequence identity, moreover, was scored against heterogeneous reference sequences and cannot be used to compare strains, so functional potency must be established by growth-phase-matched, adequately powered assays rather than inferred from the genome. These considerations define a strain-resolved, mechanism-anchored development path: controlled validation of lipid-handling and folate output, ecosystem-level testing of choline handling and TMAO attenuation, redox and inflammation biomarkers, and persistence profiling. Beyond cataloguing sequence similarity, combining genomic and functional data supports two inferences that neither approach yields alone. First, the discordance between uniformly high *bsh* identity and order-of-magnitude differences in cholesterol reduction shows that genomic screening alone is insufficient for strain selection within a species panel. Second, the concurrent absence of *cutC* and *cntA* across all three genomes, verified against assemblies of high completeness, supports a safety-relevant negative claim that phenotypic testing alone cannot establish, since the absence of TMA production under one set of culture conditions would not exclude the capacity under others. Whether the three strains together provide an advantage over the individual strains remains to be established, and is the object of the planned clinical evaluation.

## 5. Conclusions

This genome-to-function characterization shows that BR03, LRE11 and LP14 carry distinct rather than overlapping genomic repertoires relevant to cardiovascular health. All three strains carry the *bsh* gene and reduced cholesterol from the medium in vitro to strain-dependent extents; none harbored the TMA-generating genes *cutC* or *cntA*, and LP14 uniquely combines putative choline-uptake components and a choline kinase homologue, near-complete folate biosynthesis, and an enriched antioxidant and adhesion gene repertoire. Together, these complementary profiles, spanning lipid metabolism, oxidative stress mitigation, gut colonization, and choline handling, provide a coherent, mechanism-based rationale for combining the three strains into a single formulation rather than deploying them individually. This rationale generates a hypothesis for testing the combination; it does not establish that the combination is superior to the individual strains, which remains to be evaluated. The lower cholesterol reduction in LP14 relative to BR03 and LRE11, despite comparable *bsh* carriage, indicates that functional potency cannot be inferred from genome content and must be established directly; this, together with direct quantification of folate output and host-relevant validation of the proposed choline-handling effect on TMAO, defines the priorities for follow-up work. These genomic and in vitro findings have directly informed the selection criteria for a prospective randomized clinical study in patients with coronary and peripheral arterial disease, in which vascular and cardiometabolic biomarker endpoints will test whether the mechanistic rationale outlined here translates into clinical benefit.

## Figures and Tables

**Figure 1:**
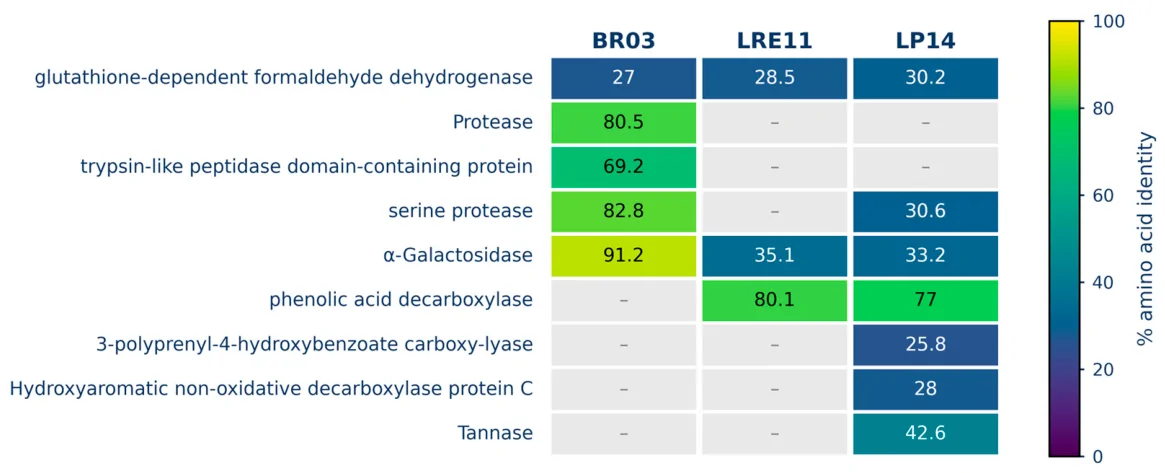
Heatmap of percentage sequence identity for anti-nutritional factor (ANF) degradation-related genes across the three probiotic strains (BR03, LRE11, LP14). Color intensity indicates percent identity to reference sequences (see Table 1).

**Figure 2:**
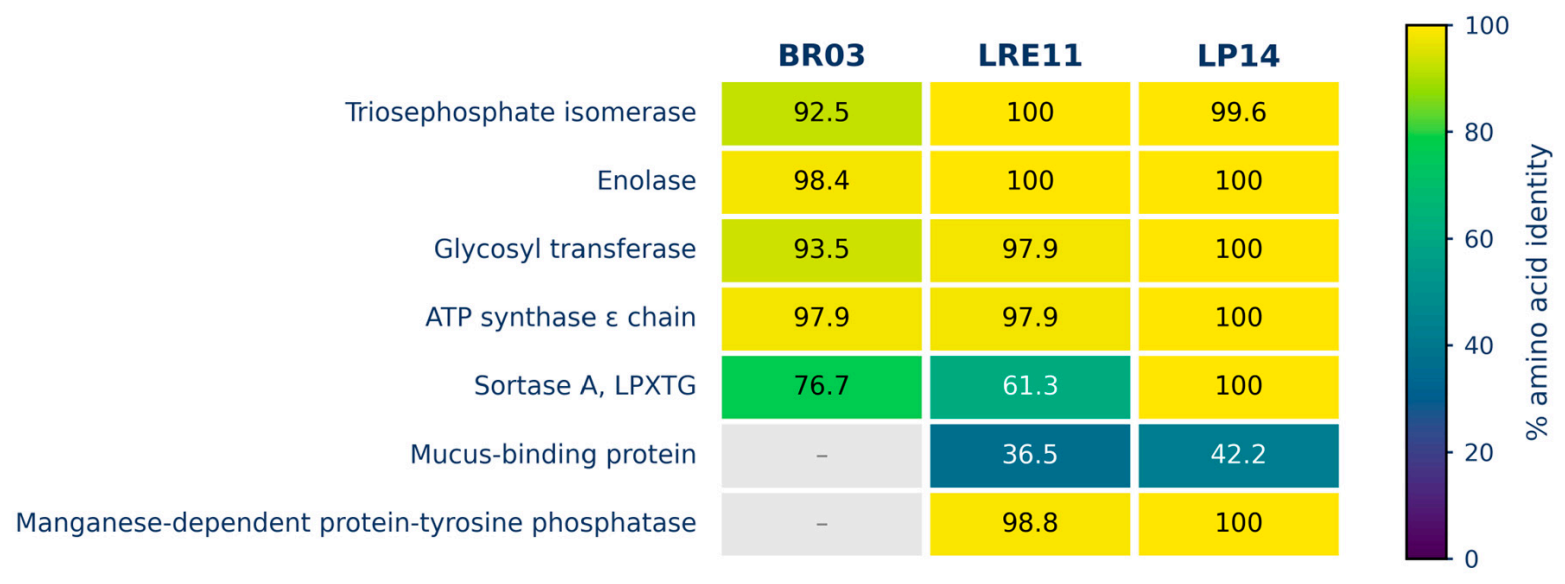
Heatmap of percentage sequence identity for adhesion-related genes across the three probiotic strains (BR03, LRE11, LP14). Color intensity indicates percent identity to reference sequences (see Table 2).

**Figure 3:**
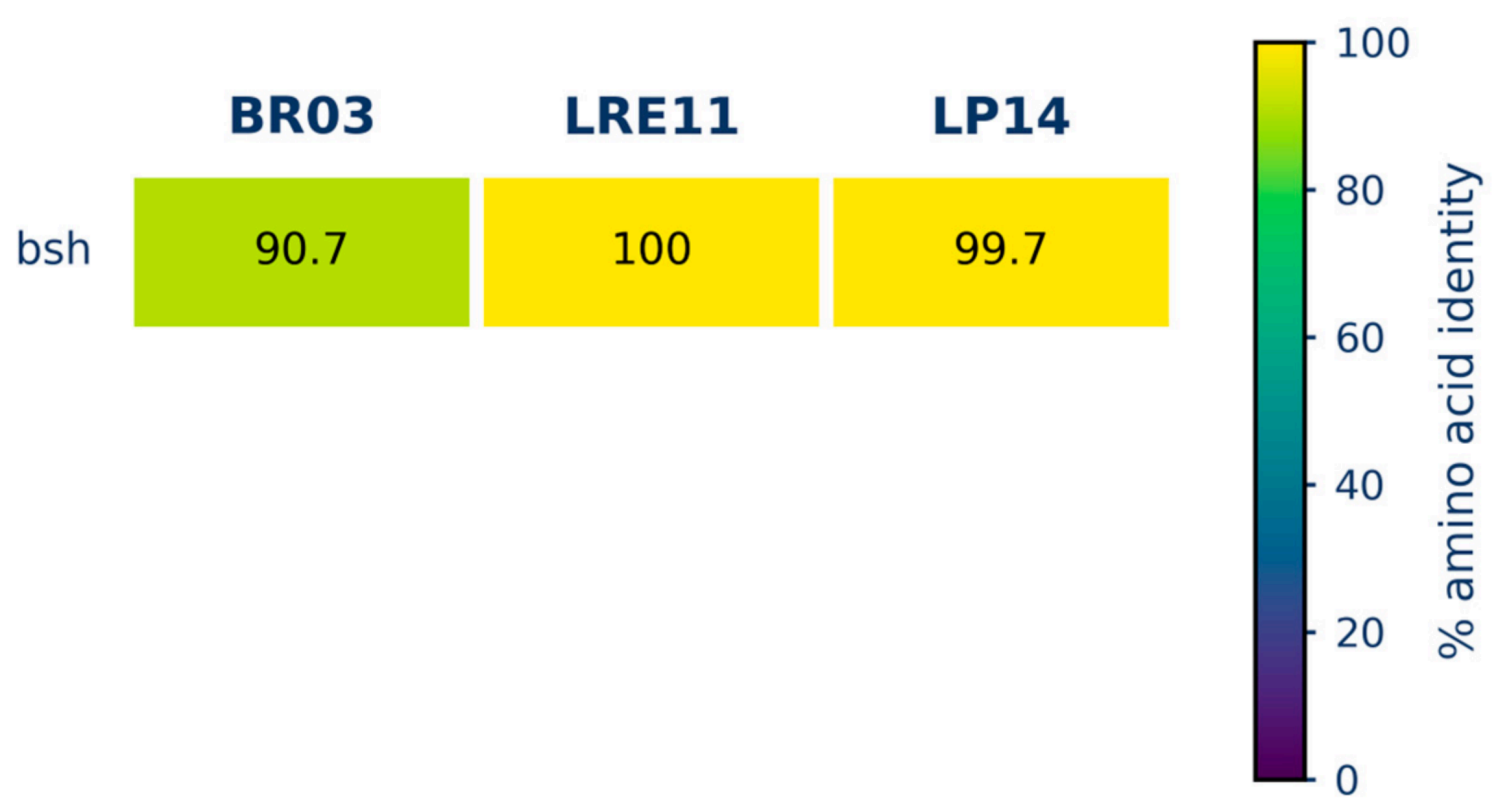
Heatmap of percentage sequence identity for the *bsh* gene across the three probiotic strains (BR03, LRE11, LP14). Color intensity indicates percent identity to reference sequences (see Table 3).

**Figure 4:**
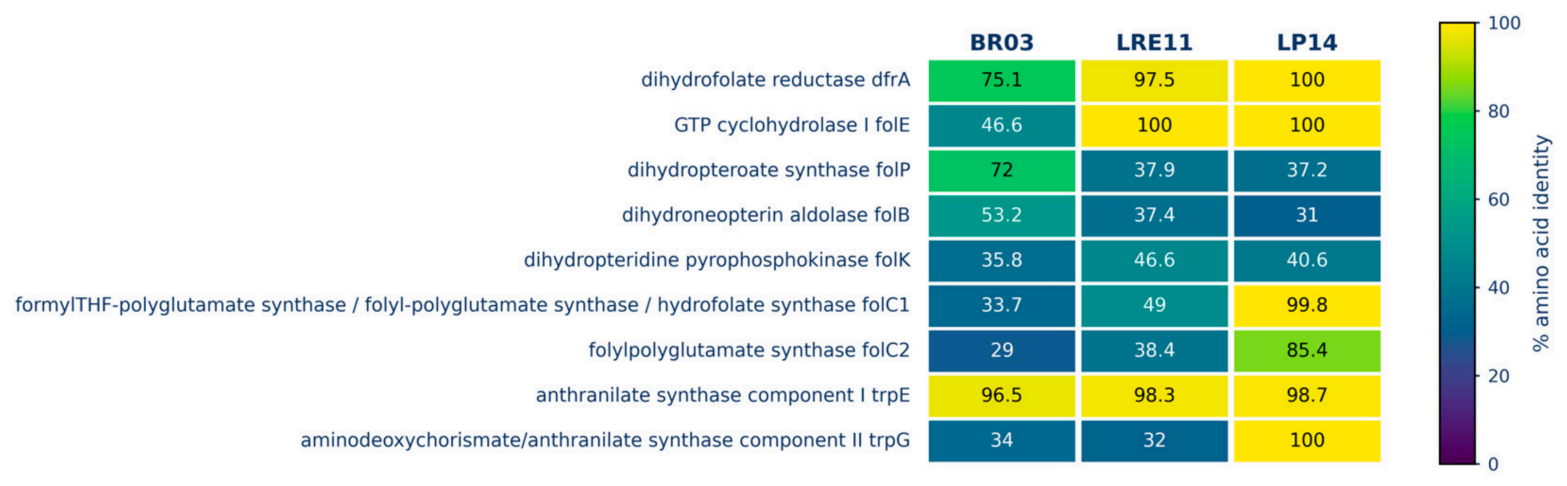
Heatmap of percentage sequence identity for folate biosynthesis-related genes across the three probiotic strains (BR03, LRE11, LP14). Color intensity indicates percent identity to reference sequences (see Table 4).

**Figure 5:**
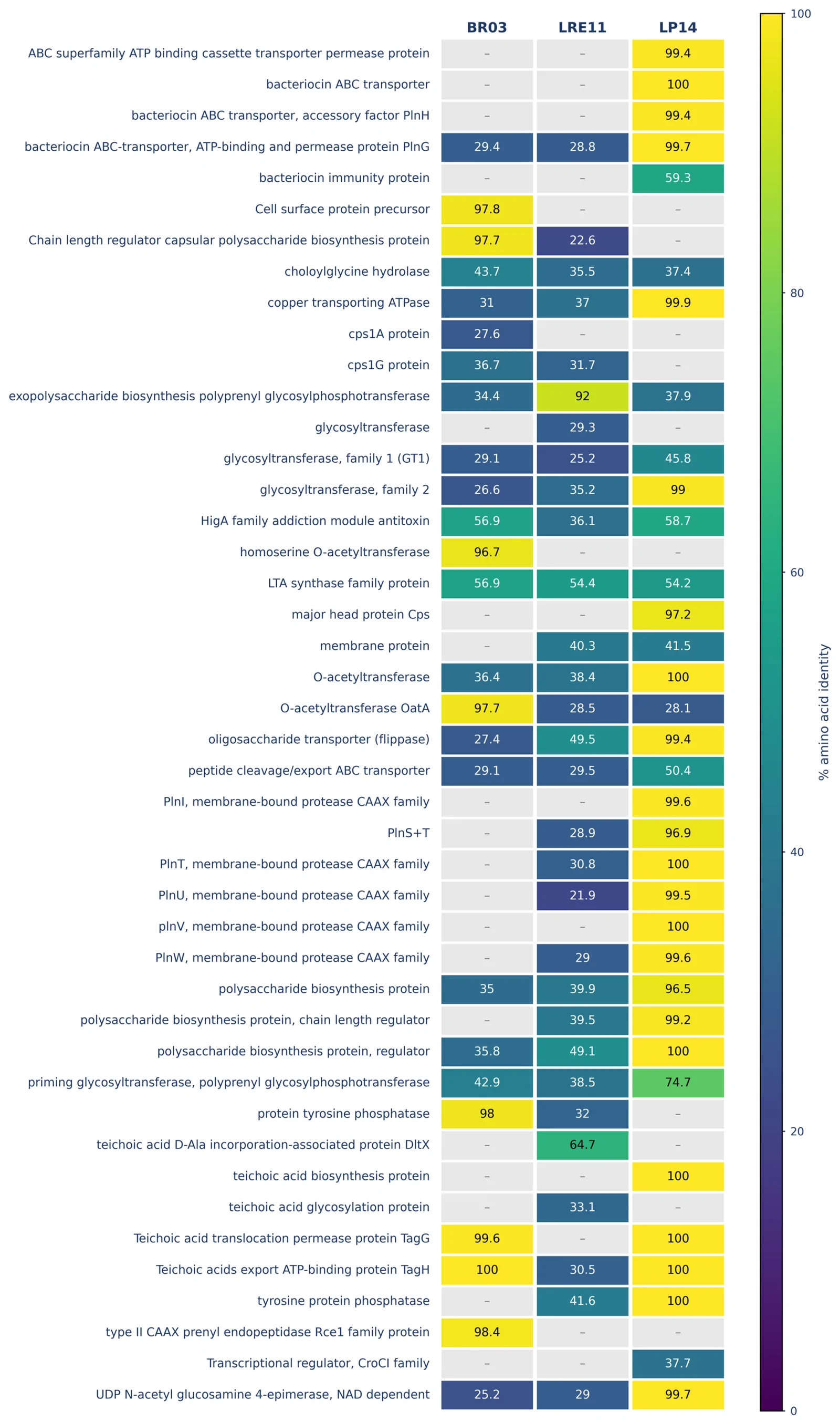
Heatmap of percentage sequence identity for immunomodulation-related genes across the three probiotic strains (BR03, LRE11, LP14). Color intensity indicates percent identity to reference sequences (see Table 5).

**Figure 6:**
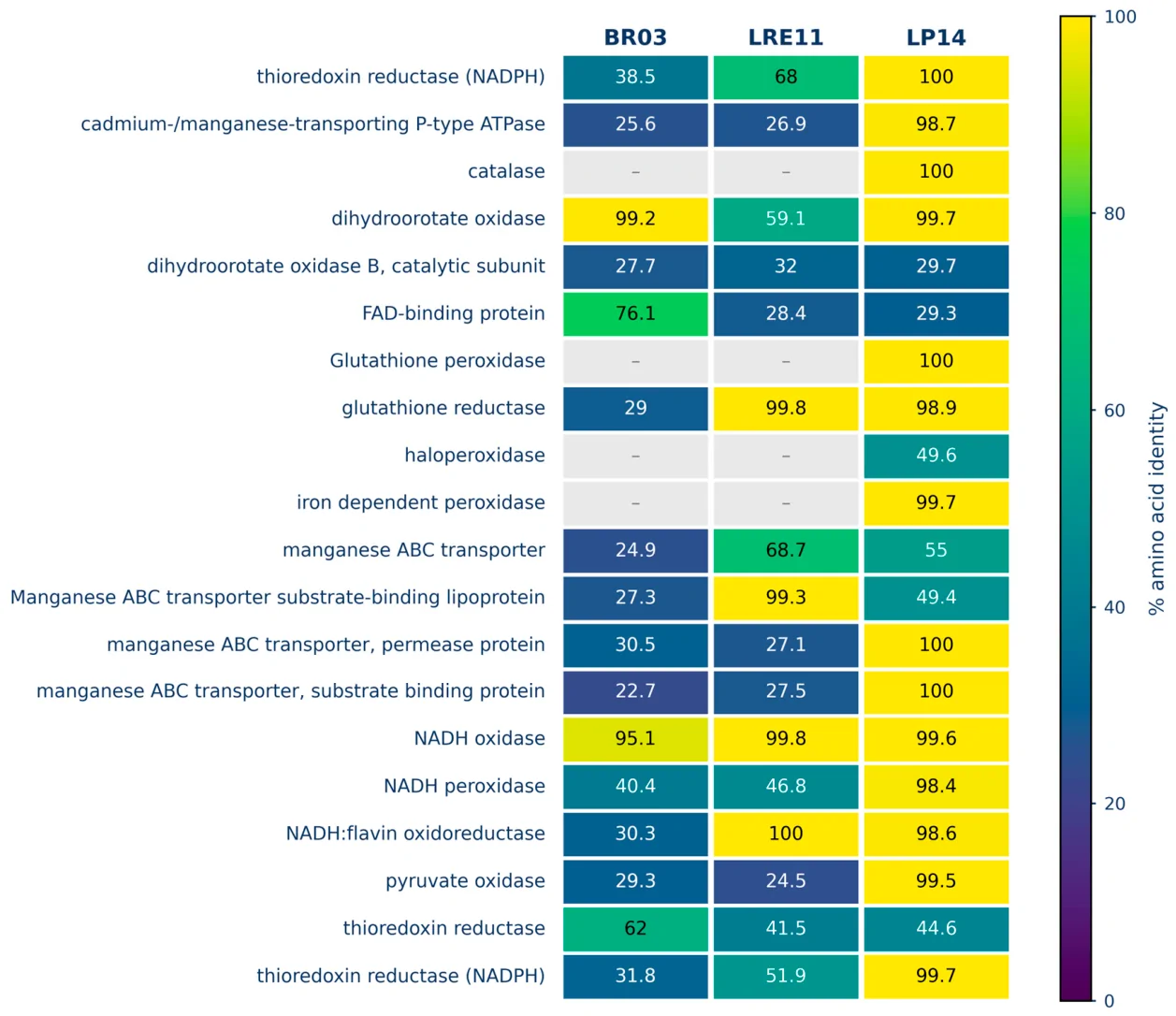
Heatmap of percentage sequence identity for oxidative stress-related genes across the three probiotic strains (BR03, LRE11, LP14). Color intensity indicates percent identity to reference sequences (see Table 6).

**Figure 7:**
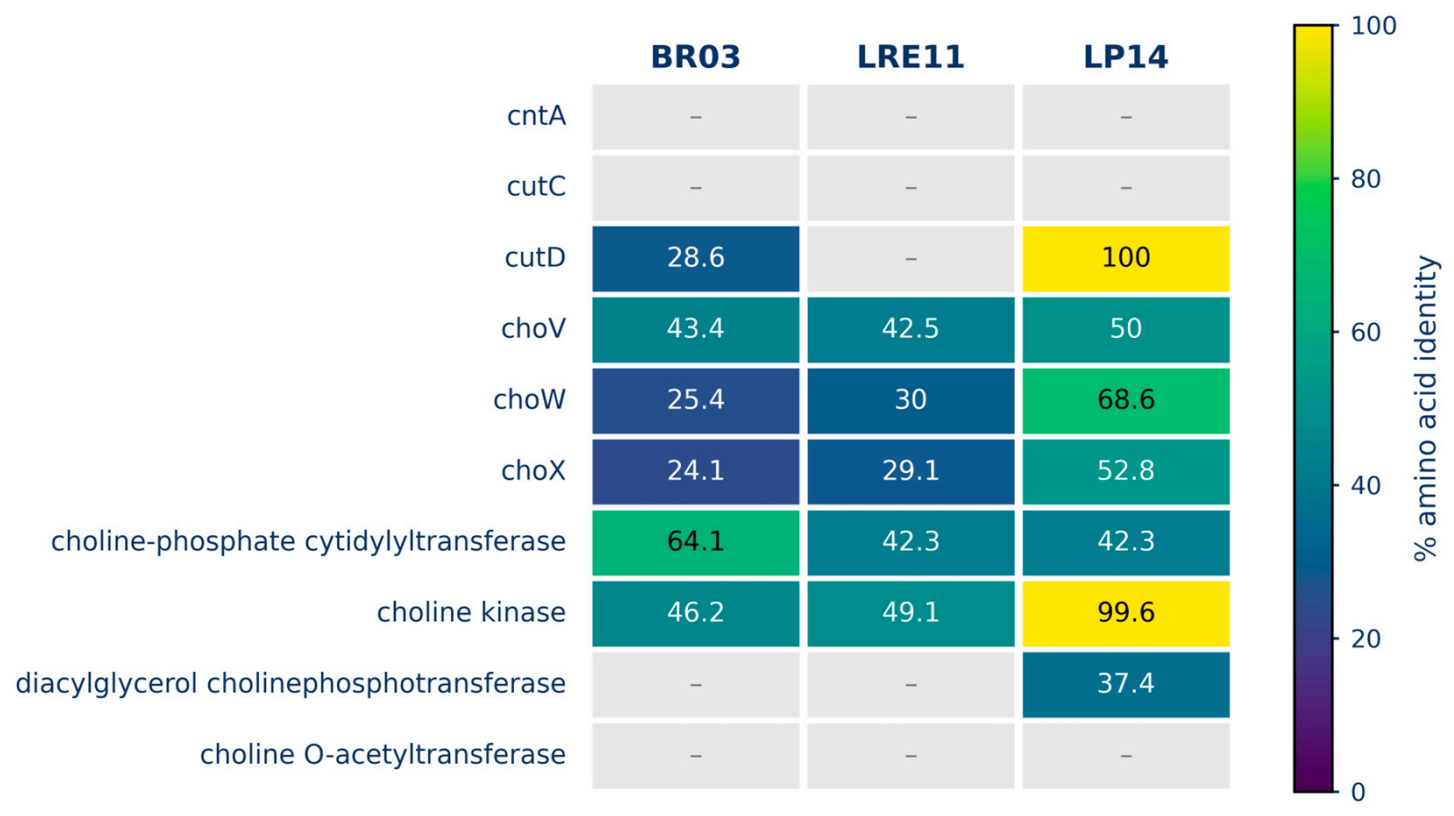
Heatmap of percentage sequence identity for choline uptake and conversion genes across the three probiotic strains (BR03, LRE11, LP14). Color intensity indicates percent identity to reference sequences (see Table 7).

**Table 1:** Percentage amino acid identity for anti-nutritional factor degradation-related genes across the three probiotic strains (BR03, LRE11, LP14).

Gene	Strains		
	BR03	LRE11	LP14
*glutathione-dependent formaldehyde dehydrogenase*	27	28.5	30.2
*Protease*	80.5	-	-
*trypsin-like peptidase domain-containing protein*	69.2	-	-
*serine protease*	82.8	-	30.6
*α-Galactosidase*	91.2	35.1	33.2
*phenolic acid decarboxylase*	-	80.1	77
*3-polyprenyl-4-hydroxybenzoate carboxy-lyase*	-	-	25.8
*Hydroxyaromatic non-oxidative decarboxylase protein C*	-	-	28
*Tannase*	-	-	42.6

Values indicate percent amino acid identity to the corresponding reference protein listed in Supplementary Table S1.

**Table 2:** Percentage amino acid identity for adhesion-related genes across the three probiotic strains (BR03, LRE11, LP14).

Gene	Strains		
	BR03	LRE11	LP14
*Triosephosphate isomerase*	92.5	100	99.6
*Enolase*	98.4	100	100
*Glycosyl transferase*	93.5	97.9	100
*ATP synthase ε chain*	97.9	97.9	100
*Sortase A, LPXTG*	76.7	61.3	100
*Mucus-binding protein*	-	36.5	42.2
*Manganese-dependent protein-tyrosine phosphatase*	-	98.8	100

Values indicate percent amino acid identity to the corresponding reference protein listed in Supplementary Table S1.

**Table 3:** Percentage amino acid identity for the bsh gene across the three probiotic strains (BR03, LRE11, LP14).

Gene	Strains		
	BR03	LRE11	LP14
*bsh*	90.7	100	99.7

Values indicate percent amino acid identity to the corresponding reference protein listed in Supplementary Table S1.

**Table 4:** Percentage amino acid identity for folate biosynthesis-related genes across the three probiotic strains (BR03, LRE11, LP14).

Gene	Strains		
	BR03	LRE11	LP14
*dihydrofolate reductase dfrA*	75.1	97.5	100
*GTP cyclohydrolase I folE*	46.6	100	100
*dihydropteroate synthase folP*	72	37.9	37.2
*dihydroneopterin aldolase folB*	53.2	37.4	31
*dihydropteridine pyrophosphokinase folK*	35.8	46.6	40.6
*formylTHF-polyglutamate synthase/folyl-polyglutamate synthase/hydrofolate synthase folC1*	33.7	49	99.8
*folylpolyglutamate synthase folC2*	29	38.4	85.4
*anthranilate synthase component I trpE*	96.5	98.3	98.7
*aminodeoxychorismate/anthranilate synthase component II trpG*	34	32	100

Values indicate percent amino acid identity to the corresponding reference protein listed in Supplementary Table S1.

**Table 5:** Percentage amino acid identity for immunomodulation-related genes across the three probiotic strains (BR03, LRE11, LP14).

Gene	Strains		
	BR03	LRE11	LP14
*ABC superfamily ATP-binding cassette transporter permease protein*	-	-	99.4
*bacteriocin ABC transporter*	-	-	100
*bacteriocin ABC transporter*, *accessory factor PlnH*	-	-	99.4
*bacteriocin ABC-transporter*, *ATP-binding and permease protein PlnG*	29.4	28.8	99.7
*bacteriocin immunity protein*	-	-	59.3
*Cell surface protein precursor*	97.8	-	-
*Chain length regulator capsular polysaccharide biosynthesis protein*	97.7	22.6	-
*choloylglycine hydrolase*	43.7	35.5	37.4
*copper transporting ATPase*	31	37	99.9
*cps1A protein*	27.6	-	-
*cps1G protein*	36.7	31.7	-
*exopolysaccharide biosynthesis polyprenyl glycosylphosphotransferase*	34.4	92	37.9
*glycosyltransferase*	-	29.3	-
*glycosyltransferase*, *family 1 (GT1)*	29.1	25.2	45.8
*glycosyltransferase*, *family 2*	26.6	35.2	99
*HigA family addiction module antitoxin*	56.9	36.1	58.7
*homoserine O-acetyltransferase*	96.7	-	-
*LTA synthase family protein*	56.9	54.4	54.2
*major head protein Cps*	-	-	97.2
*membrane protein*	-	40.3	41.5
*O-acetyltransferase*	36.4	38.4	100
*O-acetyltransferase OatA*	97.7	28.5	28.1
*oligosaccharide transporter (flippase)*	27.4	49.5	99.4
*peptide cleavage/export ABC transporter*	29.1	29.5	50.4
*PlnI*, *membrane-bound protease CAAX family*	-	-	99.6
*PlnS+T*	-	28.9	96.9
*PlnT*, *membrane-bound protease CAAX family*	-	30.8	100
*PlnU*, *membrane-bound protease CAAX family*	-	21.9	99.5
*plnV*, *membrane-bound protease CAAX family*	-	-	100
*PlnW*, *membrane-bound protease CAAX family*	-	29	99.6
*polysaccharide biosynthesis protein*	35	39.9	96.5
*polysaccharide biosynthesis protein*, *chain length regulator*	-	39.5	99.2
*polysaccharide biosynthesis protein*, *regulator*	35.8	49.1	100
*priming glycosyltransferase*, *polyprenyl glycosylphosphotransferase*	42.9	38.5	74.7
*protein tyrosine phosphatase*	98	32	-
*teichoic acid D-Ala incorporation-associated protein DltX*	-	64.7	-
*teichoic acid biosynthesis protein*	-	-	100
*teichoic acid glycosylation protein*	-	33.1	-
*Teichoic acid translocation permease protein TagG*	99.6	-	100
*Teichoic acids export ATP-binding protein TagH*	100	30.5	100
*tyrosine protein phosphatase*	-	41.6	100
*type II CAAX prenyl endopeptidase Rce1 family protein*	98.4	-	-
*Transcriptional regulator*, *CroCI family*	-	-	37.7
*UDP N-acetyl glucosamine 4-epimerase*, *NAD-dependent*	25.2	29	99.7

Values indicate percent amino acid identity to the corresponding reference protein listed in Supplementary Table S1.

**Table 6:** Percentage amino acid identity for oxidative stress-related genes across the three probiotic strains (BR03, LRE11, LP14).

Gene	Strains		
	BR03	LRE11	LP14
*thioredoxin reductase (NADPH)*	38.5	68	100
*cadmium-/manganese-transporting P-type ATPase*	25.6	26.9	98.7
*catalase*	-	-	100
*dihydroorotate oxidase*	99.2	59.1	99.7
*dihydroorotate oxidase B*, *catalytic subunit*	27.7	32	29.7
*FAD-binding protein*	76.1	28.4	29.3
*Glutathione peroxidase*	-	-	100
*glutathione reductase*	29	99.8	98.9
*haloperoxidase*	-	-	49.6
*iron-dependent peroxidase*	-	-	99.7
*manganese ABC transporter*	24.9	68.7	55
*Manganese ABC transporter substrate-binding lipoprotein*	27.3	99.3	49.4
*manganese ABC transporter*, *permease protein*	30.5	27.1	100
*manganese ABC transporter*, *substrate-binding protein*	22.7	27.5	100
*NADH oxidase*	95.1	99.8	99.6
*NADH peroxidase*	40.4	46.8	98.4
*NADH:flavin oxidoreductase*	30.3	100	98.6
*pyruvate oxidase*	29.3	24.5	99.5
*thioredoxin reductase*	62	41.5	44.6
*thioredoxin reductase (NADPH)*	31.8	51.9	99.7

Values indicate percent amino acid identity to the corresponding reference protein listed in Supplementary Table S1.

**Table 7:** Percentage amino acid identity for choline uptake and conversion-related genes across the three probiotic strains (BR03, LRE11, LP14).

Gene	Strains		
	BR03	LRE11	LP14
*cntA*	-	-	-
*cutC*	-	-	-
*cutD*	28.6	-	100
*choV*	43.4	42.5	50
*choW*	25.4	30	68.6
*choX*	24.1	29.1	52.8
*choline-phosphate cytidylyltransferase*	64.1	42.3	42.3
*choline kinase*	46.2	49.1	99.6
*diacylglycerol cholinephosphotransferase*	-	-	37.4
*choline O-acetyltransferase*	-	-	-

Values indicate percent amino acid identity to the corresponding reference protein listed in Supplementary Table S1.

**Table 8:** Cholesterol reduction by strain after incubation in MRS supplemented with cholesterol-PEG 600 (0.1 g/L), normalized to viable counts.

Strain	Cholesterol Reduction (µg per 10^9^ AFU) ± SEM
BR03	426.8 ± 8.3
LRE11	330.4 ± 100
LP14	18.9 ± 2.55

Values are expressed as µg cholesterol removed per 10^9^ AFU.

## Data Availability

The genome sequence of *B. breve* BR03 is publicly available in GenBank under accession number CP034770.1. The genome sequences of *L. plantarum* LP14 and *L. reuteri* LRE11 are proprietary to Probiotical S.p.A. and are not publicly available due to commercial confidentiality but may be made available by the corresponding author upon reasonable request. Assembly and annotation statistics are provided in Supplementary Table S2, and accession numbers of all reference protein sequences used in the alignment analysis are provided in Supplementary Table S1.

## Supplementary Materials

The following supporting information can be downloaded at https://www.mdpi.com/article/10.3390/genes17101214/s1, Table S1: Accession numbers of reference protein sequences used to build the alignment database; Table S2: Sequencing, assembly and completeness statistics of the three genome sequences.

## Abbreviations

CVD	Cardiovascular disease
TMA	Trimethylamine
TMAO	Trimethylamine N-oxide
FMO	Flavin monooxygenase
DMB	3,3-dimethyl-1-butanol
WGS	Whole-genome sequencing
*bsh*	Bile salt hydrolase
ANF	Antinutritional factor
ROS	Reactive oxygen species
AFU	Alive fluorescent unit(s)
CK	Choline kinase
PCYT	Phosphocholine cytidylyltransferase
CPT	Cholinephosphotransferase
DSM	Deutsche Sammlung von Mikroorganismen (strain collection code)
